# Associations between objectively measured physical activity intensities and executive function in preschool children: a cross-sectional study

**DOI:** 10.3389/fpsyt.2026.1900022

**Published:** 2026-09-03

**Authors:** Xin Xiong, Jiawei Lai, Siming Wang, Jian Li, Xiaohui Pei, Jiayi Sun, Yaqi An, Zhenguo Wang

**Affiliations:** 1 Department of Sports, Guangzhou Polytechnic of Sports, Guangzhou, China; 2 School of Sports Training, Guangzhou Sport University, Guangzhou, China; 3 Asset Management Office, Guangzhou Sport University, Guangzhou, China; 4 Experimental School of Fangcun Primary School in Liwan District, Guangzhou, China; 5 School of Sports, Guangzhou College of Commerce, Guangzhou, China; 6 School of Culture and Tourism, Guangdong Vocational College of Arts, Foshan, China; 7 School of Physical Education, Guangzhou Sport University, Guangzhou, China

**Keywords:** accelerometry, cognitive flexibility, executive function, inhibitory control, physical activity, preschool children

## Abstract

**Background:**

Executive function, including inhibitory control, working memory, and cognitive flexibility, develops rapidly during the preschool years and is central to children’s cognitive, behavioral, and social development. Physical activity is a potentially modifiable factor that may support executive function; however, evidence regarding the associations of specific physical activity intensities with individual executive function domains in preschool children remains limited and inconsistent. This cross-sectional study examined associations between objectively measured physical activity intensity and executive function in preschool children.

**Methods:**

The analysis included 238 children aged 3–6 years (124 boys and 114 girls; mean age, 5.3 years) from three kindergartens in Guangzhou, China. Physical activity was measured for four consecutive days using a wrist-worn ActiGraph wGT3X-BT accelerometer. Executive function was assessed using the Go/No-Go, Flanker, 1-back, and Dimensional Change Card Sort (DCCS) tasks, which indexed response inhibition, interference control, working memory, and cognitive flexibility, respectively. Group comparisons, correlation and Mantel analyses, and multiple linear regression models adjusted for age, sex, and body mass index were used.

**Results:**

Executive function performance differed across age groups in all tasks, with older children generally showing higher accuracy and shorter reaction times. Moderate-to-vigorous physical activity (MVPA), average daily MVPA, and the proportions of moderate physical activity and vigorous physical activity (VPA) also increased with age. In adjusted regression models, higher MVPA was nominally associated with shorter DCCS reaction time (*B* = −1.950, 95% CI, −3.681 to −0.219; β = −0.143; *p* = 0.027), but this association did not remain statistically significant after false discovery rate (FDR) correction (*q* = 0.108). VPA was associated with shorter Flanker reaction time before multiple-comparison correction (*p* = 0.021), but the association did not remain significant after FDR correction (*q* = 0.084). No consistent independent associations were observed between sedentary time, light physical activity, or other physical activity indicators and the remaining executive function outcomes.

**Conclusion:**

Higher-intensity physical activity showed small, task-specific nominal associations with executive function reaction-time outcomes, but these associations were not robust after FDR correction. Exploratory moderation analysis suggested that the association between MVPA and cognitive flexibility varied according to age. Given the cross-sectional design, these findings should be interpreted as tentative, domain-specific patterns rather than robust or causal effects.

## 1 Introduction

Executive function (EF) is a core component of cognitive and socioemotional development in early childhood, supporting self-regulation, goal-directed behavior, and adaptive learning (1). In China, recent policies on child development, preschool education, and healthy schools have emphasized the coordinated development of physical and mental health in preschool children (2–6). Identifying modifiable behavioral factors associated with EF during this developmental period is therefore relevant to both public health and early education. Recent research has increasingly adopted a holistic 24-h movement-behavior framework, in which physical activity (PA), sedentary behavior (SB), and sleep are viewed as interdependent behaviors that collectively influence child development (7, 8). However, the extent to which intensity-specific PA, when considered within this integrative paradigm, relates to EF in preschoolers remains underexplored.

EF is commonly conceptualized within the unity–diversity framework, in which inhibitory control, working memory, and cognitive flexibility share a common control capacity while retaining domain-specific features (9, 10). During the preschool years, however, these components remain only partially differentiated, and their structure may vary with age, task selection, and the treatment of attentional control (11–13). This rapid behavioral development parallels the maturation of the prefrontal cortex and its connections with parietal and cingulate regions. Synaptic reorganization, pruning, myelination, and increasing functional integration contribute to more efficient cognitive-control networks, with preschool children gradually showing more focal and adult-like activation patterns (14–16).

The prefrontal cortex and anterior cingulate cortex are central neural substrates of EF (17, 18). Environmental and behavioral exposures, including PA, may shape brain development and self-regulatory processes in children (19). Regular moderate-to-vigorous physical activity (MVPA) has been associated with cognitive and brain-health benefits, potentially through neurotrophic, cerebrovascular, and neurotransmitter pathways (20). Acutely, PA may increase catecholaminergic neurotransmission, physiological arousal, cerebral blood flow, and prefrontal oxygenation, thereby facilitating the allocation of neural resources during executive-control tasks. Repeated participation may also modulate neurotrophic factors, particularly brain-derived neurotrophic factor and insulin-like growth factor 1, and promote cerebrovascular adaptations that support neural plasticity and the functional efficiency of frontoparietal and cingulo-opercular networks (21, 22). Direct biomarker and neuroimaging evidence in preschool children remains limited, however, and these pathways should be regarded as biologically plausible mechanisms rather than established mediators. The preschool years represent a critical period of rapid structural and functional brain development, during which the prefrontal cortex and anterior cingulate cortex—key regions involved in attentional control, inhibitory regulation, and working memory—continue to mature (15). Given the strong experience-dependent nature and plasticity of neural networks during this stage, early exposure to physical activities, play, and SBs can significantly influence the development of cognitive control systems. PA not only enhances physiological arousal and perception of motor stimuli but also involves cognitive demands such as rule adherence, goal maintenance, movement transitions, and peer interaction, thereby repeatedly engaging executive-control processes supported by the prefrontal and anterior cingulate cortices (23). Conversely, excessive SB or screen exposure may limit children’s opportunities for physical exploration, complex play, and social interaction, thereby restricting stimuli essential for EF development. Recent applied studies in preschoolers have demonstrated that higher levels of PA, particularly participation in activities requiring coordination and cognitive engagement, are associated with improved attentional performance, inhibitory control, and working memory capacity (24). However, current evidence remains limited by factors such as study design, measurement methods, and individual variability, necessitating further validation through longitudinal and randomized intervention studies to elucidate the precise mechanisms linking PA, neurodevelopment, and cognitive outcomes.

Although accumulating evidence has indicated positive associations between PA and EF in school-aged children and adolescents (25–32), these findings cannot necessarily be generalized to preschool children. Between 3 and 6 years of age, inhibitory control, working memory, and cognitive flexibility undergo rapid but uneven development, while children’s PA is typically intermittent, play-based, and highly dependent on developmental and environmental contexts. Emerging preschool evidence suggests that associations between PA and EF may differ according to both activity intensity and EF subdomain (33). Light physical activity (LPA) generally shows weaker or less consistent associations, whereas MVPA appears more consistently related to inhibitory control and, in some studies, working memory and cognitive flexibility.

Movement behaviors should also be considered within an integrated 24-h framework, because PA, SB, screen time, and sleep co-occur and compete for time rather than operating independently. Recent observational and applied studies in preschool children illustrate this complexity. For example, Ltifi and colleagues documented contextual differences in 24-h movement behaviors, reported that habitual napping was associated with better working memory but not inhibition, and examined the potentially domain-specific effects of a structured mini-trampoline program on inhibitory control and motor skills (34, 35). More recent work has simultaneously assessed PA, SB, sleep, motor skills, and EF, while also demonstrating that these associations may be limited, domain-specific, and influenced by sex and developmental characteristics (36). Collectively, these findings support a developmentally sensitive analytical approach that distinguishes LPA, moderate physical activity (MPA), and vigorous physical activity (VPA); examines individual EF subdomains; and accounts for the combined pattern of movement behaviors across the day.

A further limitation of the existing literature is the widespread reliance on questionnaire-based PA measures (37), which are susceptible to recall and reporting biases, particularly in the context of young children’s sporadic and intermittent activity patterns. Although a few recent studies have begun to employ accelerometer-based objective monitoring in early childhood settings (38, 39), the methodological justification for intensity cut-points and the ecological validity of laboratory-based EF tasks require further clarification in preschool populations. To overcome these measurement constraints, the present study combined objective accelerometer-derived PA data with standardized, validated EF tasks, thereby providing a more rigorous estimate of the relationships between intensity-specific PA and EF in naturalistic preschool contexts.

To address the aforementioned gaps, this cross-sectional study was designed to examine the associations between objectively measured PA intensities [sedentary time (ST), LPA, MVPA, and VPA] and three core EF domains in Chinese preschoolers. We hypothesized that (1) MVPA and VPA would be positively associated with EF performance, but these associations would be domain-specific; and (2) the magnitude of these associations would vary across EF subdomains, reflecting differential neural demands. Because age is strongly related to both movement behavior and EF during the preschool period, all associations were estimated after adjustment for age. By refining our hypotheses to reflect intensity- and domain-specific effects, we aimed to provide a more nuanced understanding of these associations.

## 2 Materials and methods

### 2.1 Study design and participants

Participants were preschool children aged 3–6 years recruited from three public kindergartens in Guangzhou, China. The kindergartens provided access to outdoor playgrounds, indoor activity rooms, and a range of PA equipment. Children usually spend more than 1 h outdoors each day. Most physical activities take the form of free play, during which children can choose activities such as running, jumping, chasing games, and ball games. These activities were generally conducted with the whole class, although children could play individually or in small groups within the larger group setting. Teachers are present throughout the activities and provide guidance, demonstrations, encouragement, and support when needed. Therefore, the PA setting examined in this study closely reflects children’s everyday experiences in a typical kindergarten environment, which helps improve the external validity and real-world relevance of the findings. Written informed consent was obtained from parents or legal guardians before participation, and children were allowed to withdraw from the study at any time. The study protocol was reviewed and approved by the Ethics Committee of Guangzhou Sport University. All procedures were conducted in accordance with the Declaration of Helsinki and relevant institutional requirements.

Measurements included anthropometric indicators, accelerometer-assessed PA, and computerized EF tasks. Inclusion criteria were as follows: children aged 3–6 years; written informed consent provided by parents or legal guardians; and completion of anthropometric, PA, and EF assessments. Exclusion criteria were as follows: diagnosed cardiovascular or respiratory disease; inability to participate in moderate-intensity PA; or incomplete accelerometer or EF data.

A total of 241 preschool children were initially enrolled. Three children were excluded because their accelerometer data did not meet the valid-wear criterion. The final analysis included 238 children, comprising 124 boys and 114 girls.

### 2.2 Executive function assessment

EF was assessed using four classic tasks (9): the 1-back task for working memory (40); the Flanker task (41) and Go/No-Go task (42) for inhibitory control; and the Dimensional Change Card Sort (DCCS) task for cognitive flexibility (43). Raw data, including participant ID, trial information, accuracy, and reaction time, were exported using E-DataAid. Accuracy and mean reaction time were calculated for each task. Missing responses and abnormal reaction times were excluded according to the predefined processing rules. A speed–accuracy joint scoring method was used (44), with higher accuracy and shorter reaction time indicating better EF. Impulsive inhibition was assessed using a Go/No-Go task containing 160 formal trials, including 128 Go trials and 32 No-Go trials, with each stimulus presented for 400 ms followed by a 1,000-ms interstimulus interval. Children repeated the Go/No-Go practice block until achieving at least 70% accuracy, after which formal-trial accuracy and reaction time were recorded. Interference inhibition was assessed using a Flanker task containing 52 practice trials and 120 formal trials under neutral, congruent, and incongruent conditions. Children identified the direction of the central fish, and formal testing began only after practice accuracy reached 80%; accuracy and reaction time were recorded for each condition. Working-memory updating was assessed using a pictorial 1-back task comprising a practice block and 48 formal trials, in which children judged whether each stimulus matched the immediately preceding stimulus.

Each 1-back stimulus was presented for 3 s, and incorrect responses or responses faster than 200 ms or slower than 3 s were excluded from the reaction-time analysis. Cognitive flexibility was assessed using the DCCS task, comprising 3 practice and 6 formal color trials, 3 practice and 6 formal shape trials, and 24 mixed-rule trials. DCCS stimuli remained on the screen until a response was made, followed by a 2-s interval, and incorrect responses or reaction times below 200 ms were treated as invalid. All tasks were administered once by trained assessors in a quiet school setting, and higher accuracy together with shorter reaction time indicated better EF performance.

Although the four computerized tasks used are well validated in preschool populations, their inherent limitations should be acknowledged. First, they assess EF under decontextualized laboratory conditions that differ from naturalistic preschool settings, and recent research suggests that real-world EF performance may not fully align with lab-based measures (34–36). Second, single-session measurement may fail to capture intra-individual variability across different times or days, whereas repeated assessments provide more stable estimates (36). To partially address these limitations, we administered multiple tasks across distinct subdomains and employed standardized protocols to minimize procedural variability. Nonetheless, ecological validity remains a constraint, and future studies would benefit from incorporating complementary approaches such as teacher- or parent-reported ratings and naturalistic behavioral observations (34, 35).

### 2.3 Physical activity assessment

Participants wore a triaxial accelerometer (ActiGraph wGT3X-BT; ActiGraph, Pensacola, FL, USA) on the left wrist for four consecutive days, including three weekdays and one weekend day. Children were instructed to wear the accelerometer for 4 days, comprising three weekdays and one weekend day. This protocol was selected to capture both structured preschool-day activity and less structured weekend activity while limiting participant burden. Previous research has indicated that three valid days with at least 7 h of daily wear can provide acceptable reliability for habitual PA in preschool children (45), while recent evidence suggests that 3 to 4 days are sufficient to achieve a reliability coefficient of 0.70 for MPA, VPA, and MVPA (46). The inclusion of three weekdays and one weekend day is also consistent with validity criteria adopted in recent accelerometer studies involving young children (47, 48). Accelerometer wear duration was set at four consecutive days (three weekdays and one weekend day). This protocol was informed by Ramos-Munell et al. (46), who demonstrated that achieving a reliability score of 0.7 for MVPA requires 3 to 4 days of monitoring in preschoolers. Given that MVPA is the primary exposure of interest in this study, the 4-day protocol provides a balance between acceptable reliability and minimizing participant burden—a particularly important consideration in young children. The inclusion of at least one weekend day ensures capture of activity patterns that may differ between structured weekday and unstructured weekend settings. We acknowledge that SB and step counts may require longer monitoring durations; however, these outcomes are not the primary focus of the present study. Wrist placement was selected due to its superior compliance and wear-time adherence in young children compared to waist placement, and its ability to capture upper-body movements that are common in preschool children’s play activities. Teachers and parents assisted with device placement and ensured that the device was worn during waking hours. The accelerometer was removed only for bathing. Wear status was checked using the device-integrated wear-time sensor and the automatic wear-time validation function in ActiLife 7.2.0. A monitoring record was considered valid when the participant provided valid data for all four required days, comprising three weekdays and one weekend day. Participants who did not meet this 4-day wear criterion were excluded from the analysis.

Accelerometer data were processed using 10-s epochs and expressed as vector magnitude counts. Triaxial activity counts were summarized as vector magnitude counts, calculated as $VM=\sqrt{X^{2}+Y^{2}+Z^{2}}$. Each valid 10-s epoch was classified as ST, LPA, MPA, or VPA. The number of epochs assigned to each intensity category was multiplied by 10 s and divided by 60 to obtain minutes per day. The percentage of valid wear time spent in each intensity category was calculated by dividing the number of epochs in that category by the total number of valid epochs and multiplying by 100. No minimum activity-bout duration was imposed. MVPA was calculated as the sum of MPA and VPA. Participant-level daily estimates were calculated by averaging the values obtained across the four valid monitoring days.

**Figure 1:**
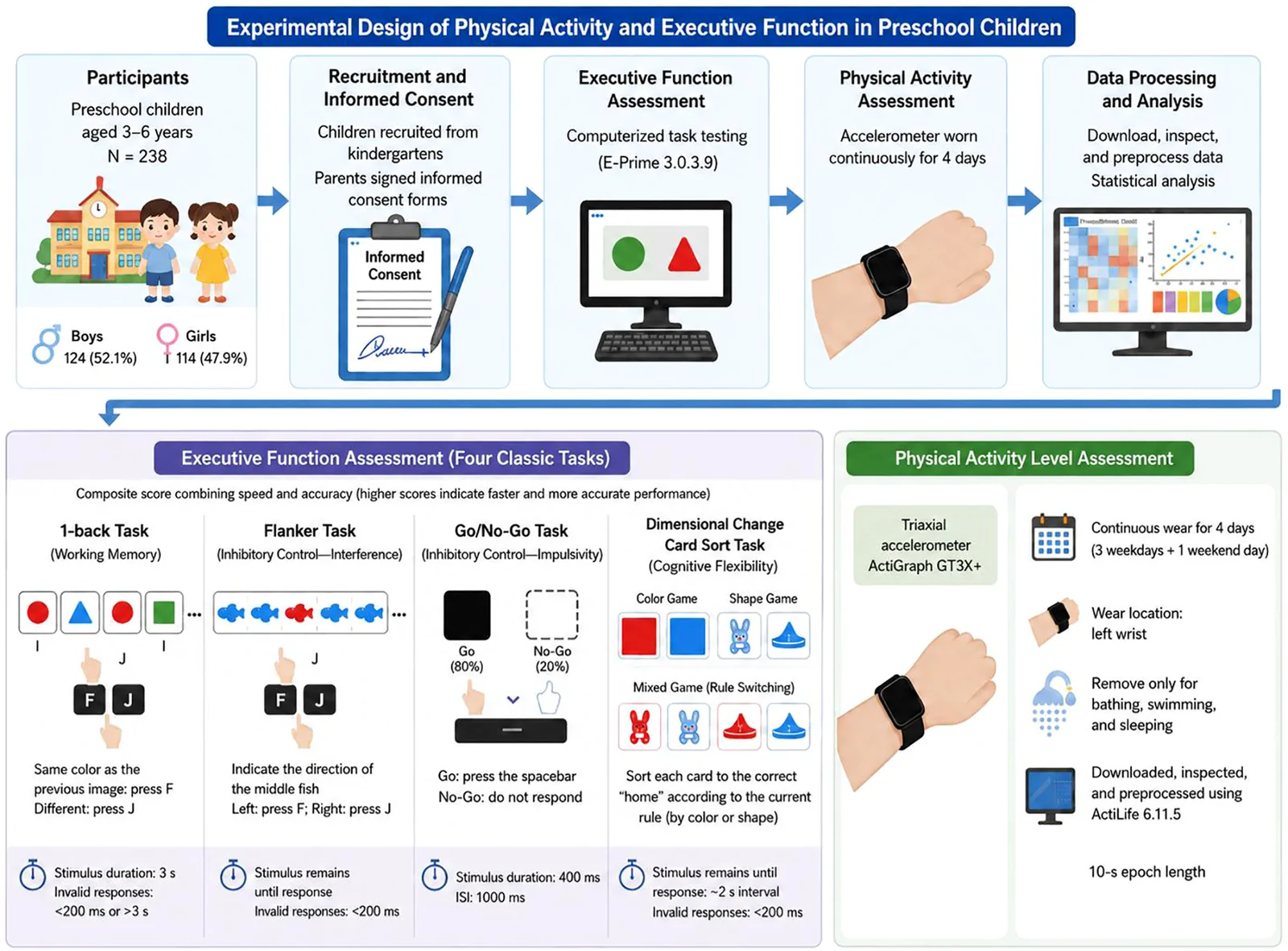
Flowchart of the experimental design for physical activity and executive function assessment.

PA intensity was classified using wrist-specific cut-points developed for preschool children by Li et al. (49). In that study, wrist-worn ActiGraph wGT3X-BT vector magnitude thresholds were reported as counts per minute. To match the 10-s epoch used in the present study, the published thresholds were converted to count-rate-equivalent 10-s values by dividing the counts-per-minute thresholds by six. Accordingly, SB was defined as ≤426 counts/10 s, LPA as 427–1,177 counts/10 s, MPA as 1,178–2,422 counts/10 s, and VPA as ≥2,423 counts/10 s. MVPA was defined as ≥1,178 counts/10 s. This conversion preserves the count-rate equivalence of the original wrist-specific thresholds but should not be interpreted as an independent validation of the thresholds at a 10-s epoch. The testing process is shown in **Figure 1**.

### 2.4 Statistical analysis

Statistical analyses were conducted using SPSS 26.0 and R 4.4.3. Categorical variables are presented as *n* (%), and continuous variables are presented as mean and standard deviation or median and interquartile range, as appropriate. The normality of continuous variables was assessed before inferential analyses. Between-sex differences were examined using independent-samples *t*-tests or Mann–Whitney *U* tests, depending on data distribution. Age-group differences were examined using one-way analysis of variance or Kruskal–Wallis tests, with *post-hoc* comparisons performed when appropriate.

Spearman correlation analyses and Mantel tests were used to explore the relationships among PA variables, covariates, and EF outcomes. The Mantel test was subsequently used to assess the overall association between the multivariate PA and cognitive-performance profiles. This method was selected because the study included multiple interrelated PA indicators and multiple EF outcomes, and the objective was to determine whether the overall pattern of between-participant differences in PA corresponded to that in cognitive performance. Before analysis, variables were standardized, and separate participant-by-participant Euclidean distance matrices were constructed for the PA and cognitive domains. The Mantel correlation coefficient was calculated between the two matrices, with statistical significance assessed using 999 permutations. The Mantel test was regarded as an exploratory global analysis of multivariate correspondence rather than an estimate of the independent effect of any individual PA variable.

Multiple linear regression models were then used to examine the associations between PA intensity and EF performance. EF outcomes were modeled separately. Age, sex, and body mass index (BMI) were included as covariates in all adjusted models. To reduce ambiguity in model interpretation, ST, LPA, MVPA, and VPA were first examined in separate regression models. Variance inflation factors were calculated to assess multicollinearity. Two-tailed *p*-values < 0.05 were considered statistically significant. To further examine the role of age, an exploratory hierarchical regression and moderation analysis was conducted for DCCS reaction time. Chronological age, sex, and BMI were entered in Model 1, MVPA was added in Model 2, and the age × MVPA interaction term was added in Model 3. Age and MVPA were mean-centered before construction of the interaction term. The incremental contribution of each step was evaluated using Δ*R*^2^ and the corresponding *F*-change test. Given that multiple linear regression models were run across three EF domains, we applied the false discovery rate (FDR) correction to control for Type I error inflation. FDR-adjusted *q*-values < 0.05 were considered statistically significant. FDR correction was applied separately within each EF task across the four PA indicators (ST, LPA, MVPA, and VPA).

## 3 Results

### 3.1 Participant characteristics

A total of 241 preschool children aged 3–6 years were initially enrolled. Three children were excluded because their PA monitoring did not meet the valid wear criterion of at least three weekdays and one weekend day. The final analysis included 238 children, comprising 124 boys (52.1%) and 114 girls (47.9%). The mean age was 5.3 years, with no significant difference between boys and girls (*p* = 0.388). No significant between-sex differences were found for height, weight, or BMI (50) (*p* > 0.05). Sex differences were observed for 1-back accuracy and DCCS accuracy (*p* < 0.05). Participant characteristics are shown in **Table 1**.

**Table 1:** Participant characteristics [mean (SD)].

Variable	Male (n = 124)	Female (n = 114)	*P*	Total (n = 238)
Age (years)	5.3 (0.9)	5.3 (0.9)	0.388	5.3 (0.9)
Height (cm)	111.7 (7.7)	112.7 (7.1)	0.273	112.2 (7.4)
Weight (kg)	20.70 (4.80)	20.77 (4.53)	0.906	20.74 (4.66)
BMI (kg/m^2^)	16.43 (2.18)	16.23 (2.33)	0.488	16.34 (2.25)
Go/No-Go accuracy (%)	87.51 (8.79)	89.09 (7.41)	0.136	88.27 (8.18)
Go/No-Go RT (ms)	589 (96)	596 (97)	0.454	592 (96)
Flanker accuracy (%)	72.61 (22.10)	74.47 (20.30)	0.500	73.5 (21.23)
Flanker RT (ms)	1,122 (254)	1,111 (221)	0.735	1,117 (238)
1-back accuracy (%)	66.49 (23.13)	72.52 (18.6)	0.028	69.38 (21.25)
1-back RT (ms)	1,420 (652)	1,473 (1162)	0.982	1,445 (930)
DCCS accuracy (%)	83.98 (11.46)	87.89 (9.28)	0.004	85.85 (10.63)
DCCS RT (ms)	1,160 (513)	1,125 (423)	0.568	1,143 (471)
ST (min)	190.67 (43.13)	192.14 (43.02)	0.793	191.37 (42.99)
LPA (min)	178.47 (34.66)	176.27 (34.53)	0.625	177.41 (34.55)
MVPA (min)	94.47 (33.98)	93.02 (35.11)	0.747	93.77 (34.46)
AD-MVPA (min/day)	95.06 (34.32)	93.61 (35.14)	0.746	94.37 (34.65)
DWT (min/day)	464.20 (60.45)	462.01 (62.74)	0.784	463.15 (61.44)
LPA (%)	40.01 (6.31)	40.27 (6.39)	0.759	40.14 (6.34)
MPA (%)	23.49 (9.70)	22.61 (9.52)	0.482	23.07 (9.60)
VPA (%)	6.01 (10.36)	6.03 (10.66)	0.985	6.02 (10.48)

RT, reaction time; ST, sedentary time; LPA, light physical activity; MVPA, moderate-to-vigorous physical activity; AD-MVPA, average daily MVPA; DWT, daily wear time; MPA, moderate physical activity; VPA, vigorous physical activity.

### 3.2 Age-group differences

When stratified by age, all EF dimensions showed significant age-related differences (*p* < 0.05). Accuracy generally increased and reaction time generally decreased with age across the Go/No-Go, Flanker, 1-back, and DCCS tasks. These findings are consistent with established developmental trajectories of EF in early childhood, reflecting the maturation of prefrontal cortical networks during the preschool period.

No significant age-group differences were found for BMI, ST, or LPA (*p* > 0.05). However, MVPA, average daily MVPA, daily wear time, and the proportions of MPA and VPA increased with age, whereas the proportion of LPA decreased with age. This pattern suggests that age may act as a developmental confounder in the association between PA and EF, as both MVPA and EF performance improve with age. We therefore included age as a covariate in all adjusted regression models to reduce the potential confounding effect of age. Detailed results are presented in **Table 2**.

**Table 2:** Age-group comparisons [mean (SD)].

Dependent variable	Age 3 (n = 22)	Age 4 (n = 52)	Age 5 (n = 88)	Age 6 (n = 76)	*F*	*P*
BMI (kg/m^2^)	15.82 (1.06)	16.00 (1.70)	16.19 (2.23)	16.74 (2.61)	1.763	0.155
Go/No-Go accuracy (%)	72.32 (15.82)	86.34 (4.90)	88.63 (6.28)	91.75 (5.13)	39.914	<0.001
Go/No-Go RT (ms)	545 (172)	613 (73)	606 (67)	592 (65)	3.409	0.018
Flanker accuracy (%)	42.88 (18.34)	60.94 (18.23)	74.88 (20.83)	84.26 (13.87)	35.259	<0.001
Flanker RT (ms)	1,249 (340)	1,252 (303)	1,089 (184)	1,047 (182)	10.969	<0.001
1-back accuracy (%)	35.04 (20.90)	62.85 (18.85)	69.51 (19.99)	78.74 (15.68)	28.838	<0.001
1-back RT (ms)	1,429 (392)	1,484 (369)	1,374 (307)	1,294 (261)	4.165	0.007
DCCS accuracy (%)	73.43 (16.66)	84.25 (12.20)	84.81 (9.58)	89.83 (6.66)	14.183	<0.001
DCCS RT (ms)	2,091 (619)	1,264 (441)	1,081 (406)	970 (262)	40.978	<0.001
ST (min)	193.45 (45.06)	181.89 (50.83)	194.75 (38.48)	192.92 (42.06)	1.001	0.393
LPA (min)	180.34 (35.12)	173.13 (41.36)	179.83 (32.93)	176.95 (32.23)	0.422	0.737
MVPA (min)	55.74 (21.41)	71.98 (33.19)	95.28 (29.51)	110.58 (29.73)	26.73	<0.001
AD-MVPA (min/day)	55.74 (21.41)	71.98 (33.19)	96.08 (29.51)	111.40 (29.80)	27.697	<0.001
DWT (min/day)	429.53 (66.97)	427.01 (91.48)	470.67 (39.99)	481.27 (45.64)	11.545	<0.001
LPA (%)	41.85 (4.67)	42.08 (5.56)	40.21 (6.48)	38.75 (6.58)	3.459	0.017
MPA (%)	13.05 (4.55)	17.74 (8.74)	23.26 (8.98)	27.46 (8.57)	21.554	<0.001
VPA (%)	0.00 (0.00)	2.52 (7.16)	6.73 (10.98)	8.28 (11.57)	5.360	0.001

RT, reaction time; ST, sedentary time; LPA, light physical activity; MVPA, moderate-to-vigorous physical activity; AD-MVPA, average daily MVPA; DWT, daily wear time; MPA, moderate physical activity; VPA, vigorous physical activity.

### 3.3 Correlation analysis

**Figure 2:**
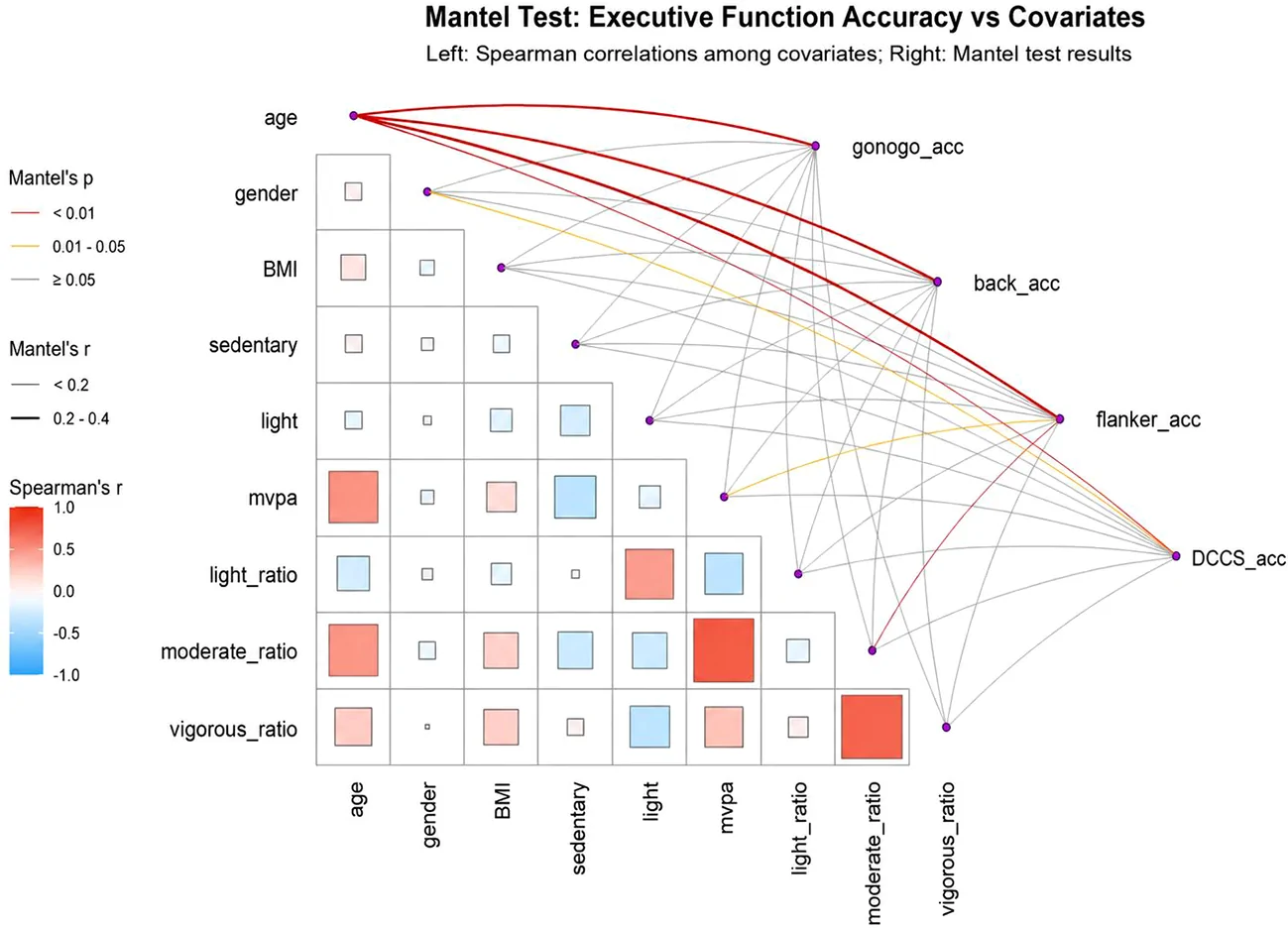
Correlation matrix between executive function accuracy and covariates.

**Figure 3:**
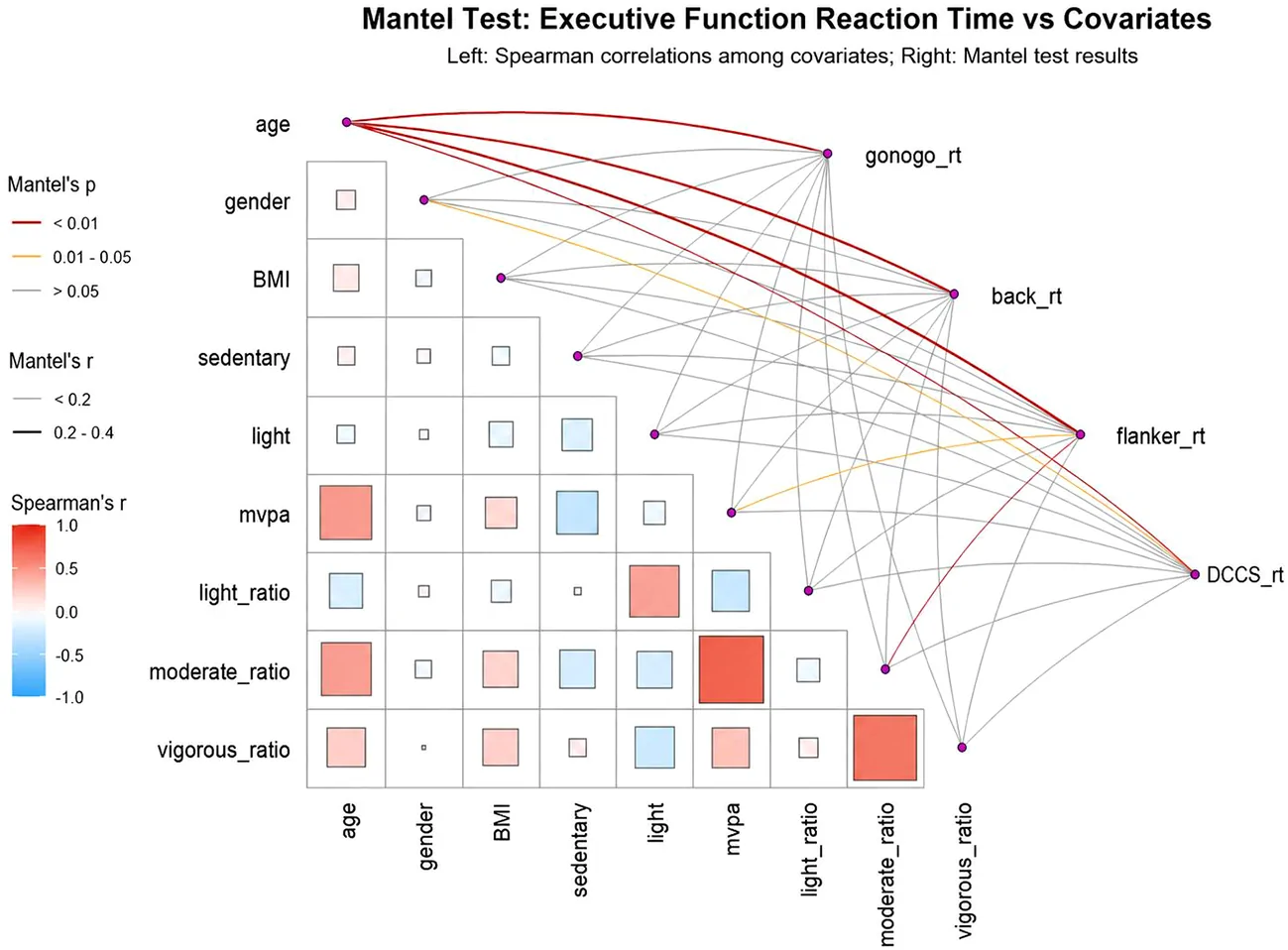
Correlation matrix between executive function reaction time and covariates.

Mantel tests showed that age was positively correlated with accuracy (**Figure 2**) and negatively correlated with reaction time (**Figure 3**) across the EF tasks (*p* < 0.01). Sex was correlated only with DCCS accuracy (*p* < 0.05). MVPA and MPA were significantly correlated with Flanker task performance (*p* < 0.05 and *p* < 0.01, respectively). ST and LPA showed weak or non-significant correlations with EF outcomes.

### 3.4 Associations between physical activity intensity and executive function

Multiple linear regression models showed that MVPA was associated with shorter DCCS reaction time (*B* = −1.950, 95% CI, −3.681 to −0.219; β = −0.143; *p* = 0.027). However, this association did not remain statistically significant after Benjamini–Hochberg FDR correction (*q* = 0.108). VPA was associated with shorter Flanker reaction time before correction for multiple comparisons (*B* = −3.71, 95% CI −6.86 to −0.56, β = −0.16, *p* = 0.021), but this association did not remain statistically significant after FDR correction (*q* = 0.084). No other associations between PA indicators and EF reaction-time outcomes were statistically significant after FDR correction. Variance inflation factors ranged from 1.071 to 1.337, indicating no substantial multicollinearity between the exposure and covariates within the fitted models. Full regression results are presented in **Table 3**.

**Table 3:** Associations between physical activity indicators and executive function reaction-time outcomes in preschool children.

Executive function domain and task	Physical activity indicator	*B* (95% CI)	Standardized β	*P*-value	FDR-adjusted *q*-value	VIF
Inhibitory control-impulsivity (Go/No-Go RT)	ST	0.26 [−1.12, 1.63]	0.03	0.715	0.841	1.071
	LPA	1.27 [−0.46, 3.00]	0.46	0.149	0.596	1.092
	MVPA	−0.63 [−2.47, 1.20]	−0.05	0.497	0.841	1.220
	VPA	−0.61 [−6.63, 5.41]	−0.01	0.841	0.841	1.217
Inhibitory control-interference (Flanker RT)	ST	−0.16 [−0.88, 0.56]	−0.03	0.667	0.667	1.071
	LPA	−0.68 [−1.58, 0.23]	−0.10	0.142	0.189	1.092
	MVPA	−0.79 [−1.75, 0.17]	−0.11	0.106	0.189	1.220
	VPA	−3.71 [−6.86, −0.56]	−0.16	0.021	0.084	1.217
Working memory (1-back RT)	ST	−2.85 [−5.71, 0.02]	−0.13	0.051	0.204	1.071
	LPA	1.83 [−1.77, 5.43]	0.07	0.317	0.634	1.092
	MVPA	−0.66 [−4.47, 3.15]	−0.02	0.734	0.820	1.22
	VPA	1.45 [−11.07, 13.96]	0.02	0.820	0.820	1.217
Cognitive flexibility (DCCS RT)	ST	−0.98 [−2.34, 0.38]	−0.09	0.155	0.310	1.071
	LPA	−0.24 [−1.95, 1.47]	−0.02	0.784	0.784	1.092
	MVPA	−1.950 (−3.681, −0.219)	−0.143	0.027	0.108	1.337
	VPA	−3.26 [−9.20, 2.69]	−0.07	0.281	0.375	1.217

RT, reaction time (ms). All models were adjusted for age, sex, and body mass index.

The standardized coefficients for the associations of MVPA with DCCS reaction time (β = −0.143) and VPA with Flanker reaction time (β = −0.160) were both small in magnitude. Although both associations were statistically significant before correction for multiple comparisons, neither remained statistically significant after FDR correction. The findings therefore suggest a tentative task-specific pattern, rather than robust evidence of consistent associations between PA intensity and EF across domains. The practical significance of these nominal associations remains uncertain, and the cross-sectional design precludes causal interpretation.

Hierarchical regression analyses were conducted to examine whether MVPA explained additional variance in cognitive flexibility performance after accounting for chronological age. In Model 1, age, sex, and BMI collectively explained 26.6% of the variance in cognitive flexibility reaction time (*R*^2^ = 0.266), with older age being significantly associated with shorter reaction time. After MVPA was added in Model 2, the explained variance increased to 28.1%, representing an additional 1.5% of variance (Δ*R*^2^ = 0.015, *p* = 0.027). Higher MVPA was associated with shorter reaction time after adjustment for age, sex, and BMI (*B* = −1.95, 95% CI: −3.681 to −0.219; standardized β = −0.143, *p* = 0.027). In Model 3, the addition of the age × MVPA interaction increased the explained variance to 32.6% (*R*^2^ = 0.326), accounting for an additional 4.5% of the variance (Δ*R*^2^ = 0.045, *p* for Δ*R*^2^ < 0.001). The age × MVPA interaction was statistically significant (*B* = 3.375, 95% CI: 1.680 to 5.070; standardized β = 0.223, *p* < 0.001). At the nominal significance level, MVPA accounted for an additional 1.5% of the variance in DCCS reaction time beyond chronological age, sex, and BMI. However, the corresponding association did not remain statistically significant after FDR correction in the primary analysis. Hierarchical regression results are presented in **Table 4**.

**Table 4:** Hierarchical regression and moderation analyses of MVPA and DCCS reaction time.

Model	Variable entered at each step	*B* (95% CI)	Standardized β	*P*	*R* ^2^	Δ*R*^2^	*p* for Δ*R*^2^	
Model 1	Age	−273.995 (−332.876, −215.114)	−0.520	<0.001	0.266			
	Sex	−5.142 (−109.376, 99.091)	−0.005	0.923				
	BMI	11.390 (−12.010, 34.790)	0.054	0.339				
Model 2	MVPA	−1.950 (−3.681, −0.219)	−0.143	0.027	0.281	0.015		0.027
Model 3	Age × MVPA	3.375 (1.680, 5.070)	0.223	<0.001	0.326	0.045		<0.001

Only variables newly entered at each step are displayed; all variables entered in preceding models were retained. Model 1 included age, sex, and body mass index. Model 2 additionally included MVPA. Model 3 additionally included the age × MVPA interaction term. All predictors entered in previous models were retained in subsequent models. Age and MVPA were mean-centered before construction of the interaction term.

## 4 Discussion

### 4.1 Main findings

This cross-sectional study examined the associations of objectively measured PA intensity with EF in preschool children. In the primary adjusted analyses, higher MVPA was nominally associated with shorter DCCS reaction time, whereas higher VPA was nominally associated with shorter Flanker reaction time. However, neither association remained statistically significant after correction for multiple comparisons. The standardized coefficients for the associations of MVPA with DCCS reaction time (β = −0.143) and VPA with Flanker reaction time (β = −0.160) were both small in magnitude. To contextualize the magnitude of this nominal association, an additional 10 min/day of MVPA corresponded to an estimated 19.5-ms shorter DCCS reaction time, equivalent to approximately 4.1% of the sample standard deviation, but its practical significance remains uncertain because the association did not survive FDR correction and no established clinically or educationally meaningful threshold for DCCS reaction time in preschool children is currently available. These findings therefore suggest tentative task-specific associations of MVPA with cognitive flexibility and of VPA with interference control, rather than robust or consistent associations between higher-intensity PA and EF across domains. Together, these findings support a cautious interpretation that higher-intensity activity may show tentative task-specific patterns of association with particular EF outcomes rather than producing a generalized cognitive benefit (25).

These findings are consistent with previous evidence suggesting that reallocating time toward MVPA may be related to better cognitive flexibility (51) and that changes in MVPA may be positively associated with cognitive flexibility (52). Consistent with the integrated movement behavior paradigm, PA, SB, and sleep should be viewed as co-dependent components of a child’s 24-h day rather than as isolated behaviors (53). Therefore, the observed association between MVPA and DCCS performance may also depend on how time is distributed across other movement behaviors, including LPA, ST, and sleep. The association between VPA and Flanker reaction time suggests that higher-intensity activity may be particularly relevant to interference-control processes. Given the cross-sectional nature of this study, these findings should be interpreted as associational rather than causal. We deliberately avoid causal language because such inferences cannot be supported by the present data. Instead, we frame these results as preliminary evidence that may inform the design of future longitudinal and experimental studies.

### 4.2 Age-related patterns

Consistent with developmental trajectories of EF (54), older children showed higher accuracy and shorter reaction time across all EF tasks. MVPA and the proportions of MPA and VPA also increased with age, whereas the proportion of LPA decreased. These age-related differences in both activity patterns and EF underscore the importance of adjusting for age when evaluating PA–EF associations in preschool children (55). The observation that both MVPA and EF improve with age further highlights the potential confounding role of developmental maturation, which we have addressed by including age as a covariate in all regression models. Hierarchical analysis showed that chronological age, sex, and BMI accounted for a substantial proportion of variance in DCCS reaction time, whereas MVPA contributed only a small additional proportion at the nominal significance level. The age × MVPA interaction was significant, but age is only a proxy for developmental maturation, and residual confounding by other factors cannot be excluded.

### 4.3 Domain- and outcome-specific patterns

The present reaction-time-specific findings may reflect differences in the measurement properties of reaction time and accuracy rather than a selective association between PA and processing speed. Accuracy is a bounded outcome and may provide limited between-child variability when performance is relatively high, whereas reaction time is continuously distributed across correct trials and may capture more subtle individual differences in processing efficiency. Nevertheless, recent intervention evidence indicates that PA can influence both reaction time and accuracy in preschool EF tasks (56). Therefore, reaction time should not be regarded as universally more sensitive than accuracy; its greater apparent sensitivity in the present study may be specific to the tasks, sample distribution, and analytical conditions.

The nominal pattern observed for cognitive flexibility, but not working memory, may reflect differences in the cognitive demands of habitual PA and the measurement properties of the tasks. However, because the association did not survive FDR correction, this domain-specific interpretation remains speculative. Many forms of preschool MVPA consist of active play, chasing games, ball activities, obstacle negotiation, and interactions with peers. These activities repeatedly require children to monitor changing environmental information, switch between rules or action plans, inhibit a previously appropriate response, and rapidly select a new movement. Such open and variable activity contexts may provide a closer cognitive match to the set-shifting demands of the DCCS than to the sustained storage and manipulation demands of working-memory tasks. Recent meta-analytic evidence indicates that cognitively engaging, coordinative, and open-skill physical activities may be especially relevant to EFs because they combine physiological exertion with rule switching, decision-making, visuospatial updating, and adaptation to unpredictable stimuli (57).

### 4.4 Potential physiological mechanisms

Several physiological pathways may underlie the observed associations between PA intensity and EF. However, these proposed mechanisms remain speculative in the present study, as we did not collect neuroimaging, electrophysiological, or biomarker data that would allow direct testing of these pathways. Accordingly, we present the following discussion as mechanistic hypotheses rather than confirmed explanations.

Several partly overlapping neurocognitive mechanisms could potentially account for the nominal patterns observed for response speed in the cognitive-flexibility and interference-control tasks. At a neurochemical level, MVPA may transiently increase catecholaminergic activity, including dopamine and norepinephrine signaling. These systems regulate arousal, attentional selection, and the signal-to-noise ratio of neural processing in prefrontal circuits. More efficient catecholaminergic modulation could enable relevant task rules to be activated more rapidly and competing responses to be suppressed with less processing delay, producing shorter reaction times without necessarily changing accuracy.

At a cerebrovascular level, PA increases cerebral perfusion and oxygen delivery. Recent fNIRS evidence in preschool children indicates that cognitively engaging PA can alter oxygenated hemoglobin concentrations in the prefrontal cortex and that these changes are associated with behavioral changes in inhibition and working memory (58). Although the present study did not measure cerebral oxygenation, enhanced prefrontal hemodynamics provide a plausible pathway through which physically active children may sustain more efficient executive processing.

At the network level, cognitive flexibility relies on the rapid reconfiguration of frontoparietal and cingulo-opercular control networks, including interactions among the dorsolateral prefrontal cortex, anterior cingulate cortex, inferior parietal regions, and striatum. These systems detect changes in task demands, maintain the currently relevant rule, disengage from the previous rule, and select an alternative response. Recent developmental neuroimaging work emphasizes that the neural systems supporting cognitive flexibility undergo substantial reorganization throughout childhood and remain particularly plastic during development (59, 60). PA may facilitate the efficiency and coordination of these networks through improved cerebral oxygenation, neurotrophic signaling, white-matter organization, and repeated engagement of perception–action switching processes.

PA may support EF by upregulating brain-derived neurotrophic factor (61), increasing cerebral blood flow, and modulating neurotransmitter systems (62), particularly in neural systems involving the prefrontal cortex. Acute exercise has also been shown to affect event-related potential indicators of cognitive control, including P3 amplitude and latency (63). These mechanisms are compatible with the nominal pattern observed between VPA and faster Flanker responses, although this association did not remain significant after FDR correction. Longer-term MVPA may contribute to structural or functional adaptations in prefrontal and hippocampal networks (64, 65), which may be relevant to cognitive flexibility.

At the nominal significance level, higher MVPA was associated with shorter DCCS reaction time, whereas higher VPA was associated with shorter Flanker reaction time. However, neither association remained statistically significant after FDR correction. These findings therefore indicate tentative, task-specific patterns rather than robust associations across EF domains. These findings suggest that higher-intensity PA may be related to selected aspects of EF performance, although longitudinal and intervention studies are needed to test causality.

### 4.5 Strengths and limitations

This study has several strengths, including objective accelerometer-based assessment, the use of multiple tasks covering distinct EF domains, and adjustment for key demographic covariates. Several considerations should nevertheless be noted when interpreting the findings. The nominal associations observed in the primary analyses did not remain statistically significant after FDR correction. Given the modest sample size and the small effect estimates, this may partly reflect limited statistical power after adjustment for multiple comparisons rather than definitive evidence of no association. This interpretation is broadly consistent with previous studies reporting positive associations between MVPA and EF; however, the present findings should still be considered tentative and require confirmation in larger, adequately powered samples. In addition, the age-moderation analysis was exploratory and requires replication. Chronological age is only an imperfect proxy for developmental maturation, and residual developmental confounding cannot be excluded. First, given the cross-sectional design, the observed associations should be interpreted as correlational rather than causal. Second, as in most accelerometry studies, estimates of activity intensity are partly influenced by the selected cut-points and data-processing procedures, although standardized protocols were applied consistently across participants. Third, computerized EF tasks provide controlled and reliable measures of specific cognitive processes, but they may not capture the full complexity of self-regulation in everyday preschool contexts. Finally, because the sample was drawn from three public kindergartens in Guangzhou, replication in more diverse cultural, socioeconomic, educational, and geographic settings would help establish the broader applicability of the findings.

## Data Availability

The original contributions presented in the study are included in the article/supplementary material. Further inquiries can be directed to the corresponding authors.

## References

[1] Diamond A . Why improving and assessing executive functions early in life is critical. In: Griffin JA McCardle P Freund LS , editors. Executive Function in Preschool-Age Children: Integrating Measurement, Neurodevelopment, and Translational Research. American Psychological Association, Washington, DC (2016). p. 11–43. doi: 10.1037/14797-002

[2] Ministry of Education of the People’s Republic of China . Guidelines for the Learning and Development of Children Aged 3–6 Years. Beijing: Capital Normal University Press (2012).

[3] State Council . Outline for the Development of Children in China (2021–2030) (2021). Available online at: https://www.gov.cn/gongbao/content/2021/content_5643262.htm (Accessed March 30, 2026).

[4] National Health Commission National Disease Control and Prevention Administration Cyberspace Administration of China National Development and Reform Commission Ministry of Education Ministry of Finance . Children and Adolescents" “Five Health” Promotion Action Plan (2026–2030) (2025). Available online at: http://www.moe.gov.cn/jyb_xxgk/moe_1777/moe_1779/202603/t20260324_1431885.html (Accessed March 30, 2026).

[5] Central Committee of the Communist Party of China State Council . Outline of the Plan for Building China Into a Leading Country in Education (2024–2035) (2025). Available online at: http://www.moe.gov.cn/jyb_xxgk/moe_1777/moe_1778/202501/t20250119_1176193.html (Accessed March 30, 2026).

[6] Ministry of Education of the People’s Republic of China . Guiding Opinions of the Ministry of Education on Comprehensively Promoting the Construction of Healthy Schools: Jiao Ti Yi [2026] No. 3 (2026). Available online at: http://www.moe.gov.cn/srcsite/A17/moe_943/moe_946/202602/t20260227_1429365.html (Accessed March 30, 2026).

[7] Tremblay MS Carson V Chaput JP Connor Gorber S Dinh T Duggan M . Canadian 24-hour movement guidelines for children and youth: an integration of physical activity, sedentary behaviour, and sleep. Appl Physiol Nutr Metab. (2016) 41:S311–27. doi: 10.1139/apnm-2016-0151 27306437

[8] Chaput JP Willumsen J Bull F Chou R Ekelund U Firth J . 2020 WHO guidelines on physical activity and sedentary behaviour for children and adolescents aged 5–17 years: summary of the evidence. Int J Behav Nutr Phys Act. (2020) 17:141. doi: 10.1186/s12966-020-01037-z 33239009 PMC7691077

[9] Miyake A Friedman NP Emerson MJ Witzki AH Howerter A Wager TD . The unity and diversity of executive functions and their contributions to complex “frontal lobe” tasks: a latent variable analysis. Cognit Psychol. (2000) 41:49–100. doi: 10.1006/cogp.1999.0734 10945922

[10] Diamond A . Executive functions. Annu Rev Psychol. (2013) 64:135–68. doi: 10.1146/annurev-psych-113011-143750 23020641 PMC4084861

[11] Deodhar AV Bertenthal BI . How attention factors into executive function in preschool children. Front Psychol. (2023) 14:1146101. doi: 10.3389/fpsyg.2023.1146101 37502749 PMC10369189

[12] Grobe SE Könen T David C Grüneisen L Dörrenbächer-Ulrich L Perels F . The factorial structure of executive functions in preschool and elementary school children and relations with intelligence. J Exp Child Psychol. (2024) 246:106014. doi: 10.1016/j.jecp.2024.106014 39043117

[13] Veraksa A Charkhabi M Aslanova M Dvorskaya E Yakupova V . Unity or diversity in executive functions: examining the three-factor model in young children. Psych J. (2025) 14:853–66. doi: 10.1002/pchj.70055 41014301 PMC12702594

[14] Fiske A Mortimer A Collins-Jones L de Klerk CCJM Gattas SU Dvergsdal H . Inhibitory control development from infancy to early childhood: a longitudinal fNIRS study. Dev Cognit Neurosci. (2025) 73:101557. doi: 10.1016/j.dcn.2025.101557 40158324 PMC11997363

[15] Eng CM Vargas RJ Fung HL Niemi SR Pocsai M Fisher AV . Prefrontal cortex intrinsic functional connectivity and executive function in early childhood and early adulthood using fNIRS. Dev Cognit Neurosci. (2025) 74:101570. doi: 10.1016/j.dcn.2025.101570 40451067 PMC12162044

[16] Varghese R Fiske A Holmboe K . Neural substrates of executive function development in children under three: a mini-review of recent advances. Front Dev Psychol. (2026) 4:1752676. doi: 10.3389/fdpys.2026.1752676

[17] Bush G Luu P Posner MI . Cognitive and emotional influences in anterior cingulate cortex. Trends Cognit Sci. (2000) 4:215–22. doi: 10.1016/S1364-6613(00)01483-2 10827444

[18] Miller EK Cohen JD . An integrative theory of prefrontal cortex function. Annu Rev Neurosci. (2001) 24:167–202. doi: 10.1146/annurev.neuro.24.1.167 11283309

[19] Mualem R Morales-Quezada L Farraj RH Shance S Bernshtein DH Cohen S . Econeurobiology and brain development in children: key factors affecting development, behavioral outcomes, and school interventions. Front Public Health. (2024) 12:1376075. doi: 10.3389/fpubh.2024.1376075 39391155 PMC11465878

[20] Hillman CH Erickson KI Kramer AF . Be smart, exercise your heart: exercise effects on brain and cognition. Nat Rev Neurosci. (2008) 9:58–65. doi: 10.1038/nrn2298 18094706

[21] Guzmán-Muñoz E Concha-Cisternas Y Jofré-Saldía E Castillo-Paredes A Molina-Márquez I Yáñez-Sepúlveda R . Physical activity and its effects on executive functions and brain outcomes in children: a narrative review. Brain Sci. (2025) 15:1238. doi: 10.3390/brainsci15111238 41300244 PMC12651547

[22] Rico-González M González-Devesa D Gómez-Carmona CD Moreno-Villanueva A . Exercise as modulator of brain-derived neurotrophic factor in children: a systematic review of randomized controlled trials. Life (Basel). (2025) 15:1147. doi: 10.3390/life15071147 40724649 PMC12300162

[23] Olive LS Telford RM Westrupp E Telford RD . Physical activity intervention improves executive function and language development during early childhood: the Active Early Learning cluster randomized controlled trial. Child Dev. (2024) 95:544–58. doi: 10.1111/cdev.14014 37800868

[24] Morales JS Alberquilla del Río E Valenzuela PL Martínez-de-Quel O . Physical activity and cognitive performance in early childhood: a systematic review and meta-analysis of randomized controlled trials. Sports Med. (2024) 54:1835–50. doi: 10.1007/s40279-024-02020-5 38598150

[25] Wang B Chen P Jia Z Tan Z . Effects of physical activity on executive function and its subdomains in children aged 5–6. Front Psychol. (2025) 16:1651806. doi: 10.3389/fpsyg.2025.1651806 41479980 PMC12753424

[26] Ziereis S Jansen P . Effects of physical activity on executive function and motor performance in children with ADHD. Res Dev Disabil. (2015) 38:181–91. doi: 10.1016/j.ridd.2014.12.005 25561359

[27] de Azevedo KPM de Oliveira Segundo VH de Medeiros GCBS de Sousa Mata AN García DÁ de Carvalho Leitão JCG . Effects of exercise on the levels of BDNF and executive function in adolescents: a protocol for systematic review and meta-analysis. Med (Baltimore). (2019) 98:e16445. doi: 10.1097/MD.0000000000016445 31305474 PMC6641795

[28] McNeill J Howard SJ Vella SA Santos R Cliff DP . Physical activity and modified organized sport among preschool children: associations with cognitive and psychosocial health. Ment Health Phys Act. (2018) 15:45–52. doi: 10.1016/j.mhpa.2018.07.001 38826717

[29] Tao Y Zhang Y Qian H Cao Z . Long-term effects of physical activity types on executive functions in school-aged children. Sci Rep. (2025) 15:30303. doi: 10.1038/s41598-025-09674-9 40830545 PMC12365328

[30] Best JR . Effects of physical activity on children’s executive function: contributions of experimental research on aerobic exercise. Dev Rev. (2010) 30:331–51. doi: 10.1016/j.dr.2010.08.001 21818169 PMC3147174

[31] Bulten R Bedard C Graham JD Cairney J . Effect of cognitively engaging physical activity on executive functions in children. Front Psychol. (2022) 13:841192. doi: 10.3389/fpsyg.2022.841192 36059731 PMC9428577

[32] Bao R Leahy AA Lubans DR Diallo TM Beauchamp MR Smith JJ . Mediators of the association between physical activity and executive functions in primary school children. J Sports Sci. (2024) 42:2029–38. doi: 10.1080/02640414.2024.2422203 39467694

[33] Luo X Herold F Ludyga S Gerber M Kamijo K Pontifex MB . Association of physical activity and fitness with executive function among preschoolers. Int J Clin Health Psychol. (2023) 23:100400. doi: 10.1016/j.ijchp.2023.100400 37663042 PMC10469079

[34] Ltifi MA Turki O Ben-Bouzaiene G Chong KH Okely AD Chelly MS . Exploring urban-rural differences in 24-h movement behaviours among Tunisian preschoolers: insights from the SUNRISE study. Sports Med Health Sci. (2025) 7:48–55. doi: 10.1016/j.smhs.2024.03.004 39649786 PMC11624334

[35] Ltifi MA Cherni Y Panaet EA Alexe CI Ben Saad H Vulpe AM . Mini-trampoline training enhances executive functions and motor skills in preschoolers. Children (Basel). (2025) 12:1405. doi: 10.3390/children12101405 41153587 PMC12563777

[36] Meksi S Ltifi MA Alfawaz W Alotaibi A Chelly MS . Sex-specific BMI, 24-hour movement behaviors, motor skills, and executive functions in Tunisian preschool children: an observational cross-sectional study. Front Physiol. (2026) 17:1853244. doi: 10.3389/fphys.2026.1853244 42245835 PMC13229776

[37] Sylvia LG Bernstein EE Hubbard JL Keating L Anderson EJ . Practical guide to measuring physical activity. J Acad Nutr Diet. (2014) 114:199–218. doi: 10.1016/j.jand.2013.09.018 24290836 PMC3915355

[38] Lau PWC Song H Song D Wang JJ Zhen S Shi L . 24-hour movement behaviors and executive functions in preschoolers: a compositional and isotemporal reallocation analysis. Child Dev. (2024) 95:e110–21. doi: 10.1111/cdev.14013 37787120

[39] Kippe K Lagestad P . The effect of activity videos on preschool children and preschool employees’ physical activity level during preschool time. Phys Act Health. (2024) 8:167–81. doi: 10.5334/paah.345

[40] Kirchner WK . Age differences in short-term retention of rapidly changing information. J Exp Psychol. (1958) 55:352–8. doi: 10.1037/h0043688 13539317

[41] Eriksen BA Eriksen CW . Effects of noise letters upon the identification of a target letter in a nonsearch task. Percept Psychophys. (1974) 16:143–9. doi: 10.3758/BF03203267

[42] Donders FC . On the speed of mental processes. Acta Psychol (Amst). (1969) 30:412–31. doi: 10.1016/0001-6918(69)90065-1 5811531

[43] Zelazo PD . The Dimensional Change Card Sort (DCCS): a method of assessing executive function in children. Nat Protoc. (2006) 1:297–301. doi: 10.1038/nprot.2006.46 17406248

[44] Shono Y Ece B Ho EH Kaat AJ LaForte EM Ayturk E . A comparison of scoring algorithms for the NIH Toolbox executive function tasks in a US norming sample. Psychol Assess. (2024) 36:760–71. doi: 10.1037/pas0001350 39666504 PMC11841212

[45] Hislop J Law J Rush R Grainger A Bulley C Reilly JJ . An investigation into the minimum accelerometry wear time for reliable estimates of habitual physical activity and definition of a standard measurement day in pre-school children. Physiol Meas. (2014) 35:2213–28. doi: 10.1088/0967-3334/35/11/2213 25340328

[46] Ramos-Munell J Antczak D Álvarez-Barbosa F Alfonso-Rosa RM del Pozo Cruz B del Pozo-Cruz J . Accelerometer monitoring duration for reliable estimates of physical activity, sedentary behavior, and step counts in preschoolers. J Phys Act Health. (2025) 22:1307–14. doi: 10.1123/jpah.2024-0640 40174888

[47] García JL Aadland E Berger N Hansen BH Benadjaoud MA van Hees V . Differences in the activity intensity distribution over the day between boys and girls aged 3 to 17 years. Sci Rep. (2025) 15:18636. doi: 10.1038/s41598-025-03866-z 40437130 PMC12120106

[48] Wang X Zhang X Yu JJ Gao W Wan M Wen X . The Goldilocks day for preschoolers’ health outcomes: a compositional data analysis of 24-h movement behaviors on weekdays and weekends. J Exerc Sci Fit. (2026) 24:100425. doi: 10.1016/j.jesf.2025.11.004 41439121 PMC12720338

[49] Li S Howard JT Sosa ET Cordova A Parra-Medina D Yin Z . Calibrating wrist-worn accelerometers for physical activity assessment in preschoolers: machine learning approaches. JMIR Form Res. (2020) 4:e16727. doi: 10.2196/16727 32667893 PMC7490672

[50] National Health Commission of the People’s Republic of China . Growth Standard for Children Under 7 Years of Age: Ws/T 423–2022. Beijing: National Health Commission of the People’s Republic of China (2022).

[51] Lu Z Qu X Chang J Xu M Song G Wang X . Reallocation of time between preschoolers’ 24-h movement behaviours and executive functions: a compositional data analysis. J Sports Sci. (2023) 41:1187–95. doi: 10.1080/02640414.2023.2260632 37724814

[52] Ishihara T Mizuno M . Effects of tennis play on executive function in 6–11-year-old children: a 12-month longitudinal study. Eur J Sport Sci. (2018) 18:741–52. doi: 10.1080/17461391.2018.1444792 29529951

[53] Chong KH Suesse T Cross PL Ryan ST Aadland E Aoko O . Pooled analysis of physical activity, sedentary behavior, and sleep among children from 33 countries. JAMA Pediatr. (2024) 178:1199–207. doi: 10.1001/jamapediatrics.2024.3330 39348138 PMC11443432

[54] Best JR Miller PH . A developmental perspective on executive function. Child Dev. (2010) 81:1641–60. doi: 10.1111/j.1467-8624.2010.01499.x 21077853 PMC3058827

[55] Kwon S O’Brien MK Welch SB Honegger K . Physical activity among US preschool-aged children: application of machine learning physical activity classification to the 2012 National Health and Nutrition Examination Survey National Youth Fitness Survey. Children (Basel). (2022) 9:1433. doi: 10.3390/children9101433 36291373 PMC9600221

[56] Liu W Wang Z Liu Y Li X . The effects of early childhood DanceSport intervention on executive function in preschool children: a randomized controlled trial. Sci Rep. (2025) 15:25003. doi: 10.1038/s41598-025-10751-2 40640487 PMC12246420

[57] Mao F Huang F Zhao S Fang Q . Effects of cognitively engaging physical activity interventions on executive function in children and adolescents: a systematic review and meta-analysis. Front Psychol. (2024) 15:1454447. doi: 10.3389/fpsyg.2024.1454447 39246315 PMC11377322

[58] Du A Ning K Shangguan C Wang C Wan B Chi A . The effects of acute cognitively engaging physical activity on executive function in preschool children: evidence from behavioral and fNIRS measures. Behav Sci (Basel). (2025) 15:1712. doi: 10.3390/bs15121712 41464055 PMC12729301

[59] Kupis LB Uddin LQ . Developmental neuroimaging of cognitive flexibility: update and future directions. Annu Rev Dev Psychol. (2023) 5:263–84. doi: 10.1146/annurev-devpsych-120221-035310 41139587

[60] Tong K Fu X Hoo NP Mun LK Vassiliu C Langley C . The development of cognitive flexibility and its implications for mental health disorders. Psychol Med. (2024) 54:3203–9. doi: 10.1017/S0033291724001508 39247963 PMC11496217

[61] Cotman CW Berchtold NC . Exercise: a behavioral intervention to enhance brain health and plasticity. Trends Neurosci. (2002) 25:295–301. doi: 10.1016/S0166-2236(02)02143-4 12086747

[62] Voss MW Nagamatsu LS Liu-Ambrose T Kramer AF . Exercise, brain, and cognition across the life span. J Appl Physiol (1985). (2011) 111:1505–13. doi: 10.1152/japplphysiol.00210.2011 21527670 PMC3220305

[63] Hillman CH Pontifex MB Raine LB Castelli DM Hall EE Kramer AF . The effect of acute treadmill walking on cognitive control and academic achievement in preadolescent children. Neuroscience. (2009) 159:1044–54. doi: 10.1016/j.neuroscience.2009.01.057 19356688 PMC2667807

[64] Erickson KI Hillman CH Kramer AF . Physical activity, brain, and cognition. Curr Opin Behav Sci. (2015) 4:27–32. doi: 10.1016/j.cobeha.2015.01.005 38826717

[65] O’Malley G . Aerobic exercise enhances executive function and academic achievement in sedentary, overweight children aged 7–11 years. J Physiother. (2011) 57:255. doi: 10.1016/S1836-9553(11)70056-X 22093124

